# Tw Cases of Labour Terminated by the Forceps

**Published:** 1854-04

**Authors:** Francis H. Ramsbotham

**Affiliations:** Obstetric Physician to the London Hospital, etc. etc.


*Tw Cases of Labour terminated by the Forceps.*—By FRANCIS H. RAMSBOTHAM, M. D. F. R. C. P., Obstetric Physician to the London Hospital, etc. etc.

I have deemed the two following forcep-cases, that have recently occurred in my practice, worthy of publication, because of the contrast which they supply; the one woman who recovered perfectly well, having been in labor for six days with her first child, and the membranes having been ruptured seventy-five hours before delivery was effected; while the other, who died, had borne nine children before, and in her case the liquor amnii had been evacuated only five hours. I may add, that in both, the head was extracted with less than the ordinary degree of difficulty.

*Case* 1.—*Forceps: Fatal.*—At 9 A. M., on Thursday, Nov. 3, 1853, I was sent for by Mr. Houghton to Mrs. W., Nelson-square, Blackfriars-road, a very stout woman, of leuco-phlegmatic temperament, weak powers, and unhealthy constitution, in labor of her tenth child. She had been taken ill during the preceding evening; and the membranes had broken at four in the morning. Since that time, however, the uterus had been acting with great power, and the attendant pain had been unusually severe. Although her husband had driven rapidly to my house, and I obeyed the summons immediately, her first exclamation when I entered the room, was, "How long you have been coming!" for she had been promised delivery as soon as I should arrive. I found the os uteri perfectly dilated, the vagina very lax, the bladder empty, a great part of the head in the pelvic cavity, the base being still above the brim, and the face turned towards the left groin. The cause of delay was evidently a contraction in the conjugate diameter of the brim; but as I judged the space to be at least three inches and a quarter, I expected to meet with little difficulty in extraction by the long forceps. The uterus was contracting very strongly, and almost incessantly; the abdomen was very pendulous, and the whole of the uterine tumour so tender, that she would not bear the least pressure upon it. The pulse was very rapid, the countenance suffused and much distressed, and there was very frequent, indeed almost constant vomiting, everything she took being instantly rejected: there was, however, no black or coffee-ground-like matter. I immediately applied the long forceps most easily, one blade being passed up to the right eye-brow, and the other behind the left ear. With two efforts I brought the head into the pelvis, and, owing to the distensibility of the soft parts, extraction was very easily accomplished. The shoulders, however, gave me considerable trouble, and I was obliged to use a blunt hook for their liberation. The child was dead; it weighed twelve pounds and a half. The placenta passed naturally, without hæmorrhage, in about ten minutes. I was in hopes that both the vomiting and the uterine tenderness would cease on delivery; but in this I was disappointed; for in neither respect did any material amendment take place. On the next day, however, she was rather better, and I began to entertain some hopes of her recovery. In consequence of being obliged to go some distance into the country on that evening, (Saturday,) I did not see her again till early on Tuesday morning. She was then in a state of most urgent danger, all the symptoms being aggravated; the vomiting was almost constant, and the egesta of a dark color; the abdomen was also tympanitic. On Wednesday she was even worse, and she died at ten o'clock on Thursday morning. No *post mortem* inspection was allowed, but I have little doubt that the uterus had injured itself by the violence of its action after the membranes broke. There were none of the local indications of laceration present, indeed; but the peritonæal covering might have split, as it sometimes, though rarely, does; or the mucous membrane, and a portion of the substance might have given way without any communication being made with the abdominal cavity, under either of which circumstances, of course, no recession would take place of any part of the child.

This is a kind of case in which, no doubt chloroform would have been useful, and I should have administered it myself, had I not been anxious to effect delivery without delay, and certain that I could do so with great facility.

*Case* 2.—*Forceps: Recovery.*—On Saturday, Dec. 30, 1853, at 10 A. M., Mr. York called me to Mrs. S., in the Edgeware-road, aged 41, in labor of her first child. Mr. York had been sent for to her on the Sunday before. She was then suffering from periodical pains, like those of labor, and they had continued, without intermission, more or less severely ever since. The membranes broke spontaneously on Wednesday morning, since which time the uterus had been acting regularly, as well as more powerfully than before; the pains, however, had never been of an expulsive character till lately. I found the os uteri almost entirely dilated, but to be felt all round, soft, thin, and distensible. The head was not fully in the pelvis; it was detained, apparently, by a want of space above the brim, in the conjugate diameter, owing to the last, or the two lowest lumbar vertebræ projecting too far forwards. The promontory of the sacrum was quite out of the reach of the finger, and yet there evidently existed some mechanical impediment, which must have been situated higher than the brim, to the head's descent. The head itself was perfectly free and unimpacted, and the face was looking towards the right groin. There was no distress in the system; the pulse was under 100; the tongue was clean, the mouth moist, the countenance placid, the urine passed freely, and the patient took a sufficiency of nourishment, and had all along obtained a good share of rest at short intervals. There was no tenderness of the uterine tumour externally, nor of the vagina within. The uterus was acting expulsively every four or five minutes, and the child's head was bearing upon the os uteri with each return of pain. I considered that it would have cleared the orifice long before the time I saw her, had it not been for the resistance noticed above.

Both the patient and her friends were very desirous that I should terminate the labor at once. Nor is this to be wondered at, considering the length of time that it had already lasted; but as delivery could only have been effected at that time by sacrificing the child, and as such a proceeding could not possibly be entertained, Mr. York assisted me in persuading them of the propriety of postponing any instrumental means for the present. I saw her again at 5.15. Both she and her friends had then become very importunate. The head was much elongated; it was somewhat distending the perinæum, and the vertex was being partially protruded with each contractile effort, though it had not completed its turn. The pains were decidedly expulsive, but still not violent. The external parts were rigid, and giving way slowly; there was no urine in the bladder; the pulse was rather more frequent, and the lady was altogether more anxious about herself than in the morning; still there were no very urgent symptoms. Taking into account, however, the length of time that had elapsed since the membranes broke, the want of progress that was then being made, (although the head had descended considerably since my first visit,) together with her own and her friends' urgent solicitations, and the ease with which delivery could be effected, we thought it better for the child's sake, as well as her own, not to allow the labor to proceed any longer. I therefore introduced the blades of the forceps upon the head laterally as regarded the pelvis, not being able to feel an ear, and extracted it in two or three minutes. It escaped quickly as soon as the turn was completely made; one limb of the instrument was adapted over the left brow, the point reaching to the orbitar ridge, and the other behind the right ear. The child was alive, and its face quite livid; it soon, however, recovered its natural color. The trunk passed easily. The umbilical cord was more than four feet in length, but was not twisted round any part of the child's body. The placenta was expelled in fifteen minutes, without hæmorrhage. The next day she was as well as if she had gone through the most natural labor; she has progressed satisfactorily, and is able to nurse her infant.

The foregoing may be taken as a strongly-marked example of two different types of cases, though terminated by the same instrument with ease; the one for the absence of constitutional distress in a labor that had lasted six days; the other, for the supervention of highly dangerous, indeed fatal symptoms, after the lapse of a few hours. They show how little time must be taken into account when agitating the question of the necessity of artificial assistance; how much more requisite it is to regard present symptoms, and to be guided by the patient's actual condition.

*Medical Times and Gazette.*

